# Use of delayed antibiotic prescription in primary care: a cross-sectional study

**DOI:** 10.1186/s12875-019-0934-7

**Published:** 2019-03-26

**Authors:** Mariam de la Poza Abad, Gemma Mas Dalmau, Ignasi Gich Saladich, Laura Martínez García, Carl Llor, Pablo Alonso-Coello

**Affiliations:** 1 Dr Carles Ribas Primary Care Center, C/Foc 112, 08038 Barcelona, Spain; 2 Iberoamerican Cochrane Center, Biomedical Research Institute Sant Pau (IIB Sant Pau), Barcelona, Spain; 3 Via Roma Primary Care Center, Barcelona, Spain; 4 CIBER Epidemiology and Public Health (CIBERESP), Barcelona, Spain

**Keywords:** Delayed antibiotic prescription, Primary care, Survey, Infectious disease

## Abstract

**Background:**

One of several strategies developed to reduce inappropriate antibiotic use in situations where the indication is not clear is delayed antibiotic prescription (DAP), defined as an antibiotic prescription issued for the patient to take only in case of feeling worse or not feeling better several days after the visit. We conducted a survey to identify DAP use in Spanish primary care settings.

**Methods:**

We surveyed 23 healthcare centers located in 4 autonomous regions where a randomized controlled trial (RCT) on DAP was underway. The primary variable was use of DAP. Categorical and quantitative variables were analyzed by means of the chi-squared test and non-parametric tests, respectively.

**Results:**

The survey was sent to 375 healthcare professionals, 215 of whom responded (57.3% response rate), with 46% of these respondents declaring that they had used DAP in routine practice before the RCT started (66.6% afterwards), mostly (91.5%) for respiratory tract infections (RTIs), followed by urinary infections (45.1%). Regarding DAP use for RTIs, the most frequent conditions were pharyngotonsillitis (88.7%), acute bronchitis (62.7%), mild chronic obstructive pulmonary disease exacerbations (59.9%), sinusitis (51.4%), and acute otitis media (45.1%). Most respondents considered that DAP reduced emergency visits (85.4%), scheduled visits (79%) and inappropriate antibiotic use (73.7%) and most also perceived patients to be generally satisfied with the DAP approach (75.6%). Having participated or not in the DAP RCT (74.1% versus 46.2%; *p* < 0.001), having previously used or not used DAP (86.8% versus 44.2%; p < 0.001), and being a physician versus being a nurse (81.8% versus 18.2%; p < 0.001) were factors that reflected significantly higher rates of DAP use.

**Conclusions:**

The majority of primary healthcare professionals in Spain do not use DAP. Those who use DAP believe that it reduces primary care visits and inappropriate antibiotic use, while maintaining patient satisfaction. Given the limited use of DAP in our setting, and given that its use is mainly limited to RTIs, DAP has considerable potential in terms of its implementation in routine practice.

**Electronic supplementary material:**

The online version of this article (10.1186/s12875-019-0934-7) contains supplementary material, which is available to authorized users.

## Background

Infectious diseases are among the most common reasons for visits to primary care centers. Approximately 70% are respiratory tract infections (RTIs), most frequently, rhinitis, pharyngitis, and acute bronchitis [1]. Most RTIs are self-limiting, with recent reviews suggesting that —except in the case of an underlying comorbidity— antibiotics offer little or no clinical benefit [2, 3]. Inappropriate prescription of antibiotics —as well as implying a cost for national health systems and fostering a false belief that antibiotics are always beneficial— has serious consequences for patients’ health, including the risk of adverse effects and antimicrobial resistance [4]. In recent years, the World Health Organization (WHO) has prioritized the problem of antimicrobial resistance in its agenda [5].

Several strategies have been developed to reduce inappropriate use of antibiotics. One of them is delayed antibiotic prescription (DAP), whereby the prescription is issued for the patient to take only in the event of feeling worse or not feeling better several days after the visit. DAP has been widely studied and applied in English-speaking countries [6], and is especially recommended as a potential strategy for treating acute uncomplicated RTIs [7]. DAP has also been shown to be effective in uncomplicated urinary tract infections [8] and in acute infective conjunctivitis [9], with better results when DAP is implemented in conjunction with appropriate and structured advice for the patient [10].

In Spain there is little information about the use of DAP. Llor et al. [11] conducted an observational study that showed that DAP resulted in reduced antibiotic use. More recently, our research group published results for a multicenter randomized controlled trial (RCT) of DAP [12] that confirmed reduced antibiotic use, similar satisfaction levels with other antibiotic strategies, and no increase in adverse effects or re-visits [13]. Since no information on use of DAP is available for our setting, despite its effectiveness in treating acute uncomplicated RTIs, we conducted a survey in primary care healthcare centers in Spain.

## Methods

### Design

Multicenter cross-sectional survey.

### Study population

Healthcare staff from 23 Spanish health centers where an RCT on DAP was being conducted [12]. The healthcare centers were located in the 4 Spanish Autonomous Regions of Catalonia, Navarra, Madrid, and the Basque Country. Included were all healthcare professionals employed in those centers regardless of whether or not they were participating in the RCT.

The selected participants were those authorized to prescribe treatments, namely, primary care physicians, medical residents and registered nurses. Nurses were taken into account, given that in Spain they are authorized to attend to initial emergency cases in primary care centers [14]. We defined respondents as all individuals who returned a filled-in questionnaire.

### Survey development

We developed the questionnaire based on a review of the scientific literature. Using a combination of descriptors and free-text terms (Additional file 1), we conducted a search in MEDLINE (via PubMed, from inception until March 2012) to identify studies of DAP.

We piloted the questionnaire with 6 healthcare professionals (2 primary care physicians, 2 nurses and 2 epidemiologists) and evaluated its sensitivity. The final questionnaire included 22 items grouped into 5 sections (Additional file 2): (1) sociodemographic data; (2) clinical scenarios; (3) awareness of and participation in the DAP RCT; (4) use of DAP; and (5) perceptions of DAP. Referring to the clinical scenarios, with the aim of assessing use of DAP in routine practice, the respondents were asked about 2 cases of uncomplicated RTIs posing clinical uncertainty regarding the prescription of antibiotics, namely, pharyngotonsillitis and chronic obstructive pulmonary disease (COPD) exacerbation. An online tool was used to run the survey and to collect responses, and 3 reminders were sent by email at 2-week intervals following initial contact.

### Analysis

The data were analyzed descriptively, with absolute frequencies and proportions calculated for categorical variables, and means and standard deviations (or median and range when normality criteria were not fulfilled) calculated for quantitative variables. Groups of categorical variables were compared using the chi-squared test, and groups of quantitative variables using analysis of variance (ANOVA) for unpaired data or non-parametric tests (Mann-Whitney).

Differences in DAP use by disease and by healthcare professional characteristics (age, occupation, and RCT participation) were analyzed by comparing proportions using the chi-squared test. Responses to open questions were analyzed and coded according to the most frequent topics. Statistical significance was set to *p* < 0.05 and data were analyzed using SPSS version 24.0 (IBM-SPSS).

## Results

A total of 375 healthcare professionals received the questionnaire, of whom 37.7% were participating in the RCT; 215 individuals replied to the questionnaire (response rate 56%). The mean age of respondents was 46.2 (10.1 SD) years, 72.6% (*n* = 156) were family physicians, and 74.4% (*n* = 160) were women. Respondent characteristics are described in Table 1.

**Table 1:** Descriptive characteristics of delayed antibiotic prescription (DAP) survey respondents

		Number	Percent
Profession	Physician	156	72.6%
	Nurse	59	27.4%
Participating center	Catalonia	115	53.5%
	Madrid	72	33.5%
	Navarra	23	10.7%
	Basque Country	5	2.3%
Teaching center	Yes	146	67.4%
	No	70	32.6%
Rapid diagnostic techniques^a^	Multistix urine test strip	194	90.2%
	Reactive Strep-A	39	18.1%
	Reactive PCR	22	10.2%
	Other	15	7%
Used DAP before RCT	Yes	99	46%
	No	57	26.5%
Used DAP during RCT	Yes	143	66.6%
	No	16	7.4%
DAP type (for DAP users)	Direct (patient-led)	106	76.3%
	Collection from reception	21	15.1%
	Referral to physician	10	7.2%
	Other	2	1.4%

^a^Multistix urine test strip (in diagnosis of urine infection), Rapid antigen detection test (Group A streptococcal in pharyngitis) and C-reactive protein (in assessing etiological diagnosis of acute respiratory infection)

Of the total respondents (*n* = 215), 46% (*n* = 99) had used DAP in routine practice before the DAP RCT (37.8% of physicians and 15.3% of nurses; *p* = 0.013), and 66.6% (*n* = 143) used DAP in routine practice during the DAP RCT (69.2% of physicians and 20.3% of nurses; *p* < 0.001). Regarding how DAP was applied, 76.3% (*n* = 106) of patients received DAP directly, 15.1% (*n* = 21) collected the prescription from reception, 7.2% (*n* = 10) were referred to their physician, and other strategies were used for 1.4% (*n* = 2) of patients.

**Fig. 1:**
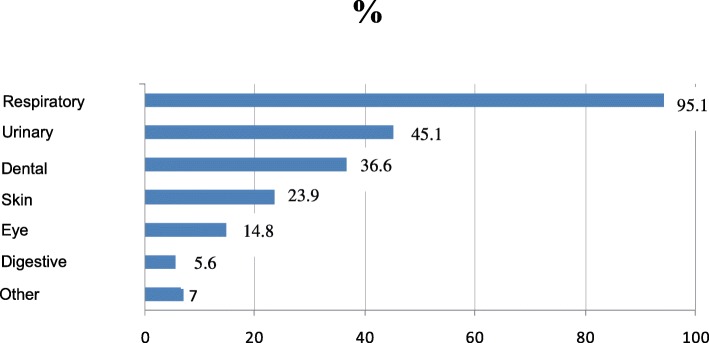
Delayed antibiotic prescription use by infection type

**Fig. 2:**
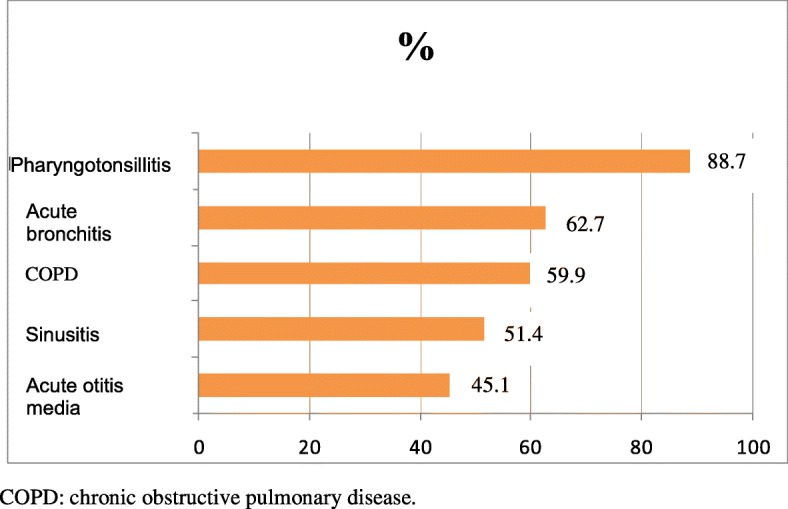
Delayed antibiotic prescription use by respiratory disease

DAP was used mainly for acute RTIs (n = 143; 91.5%), followed, at a distance, by urinary infections (45.1%), dental infections (36.6%), skin infections (23.9%), eye infections (14.8%), digestive infections (5.6%), and other infections (7%) (Fig. 1). Regarding DAP use for RTIs, the most frequent conditions were pharyngotonsillitis (88.7%), acute bronchitis (62.7%), mild COPD exacerbations (59.9%), sinusitis (51.4%), and acute otitis media (45.1%) (Fig. 2). Regarding prescription strategies for patients with pharyngotonsillitis, 50.2% received no antibiotic prescription, 3.3% immediate antibiotic prescription, and 30.7% received DAP (19.1% directly and 11.6% at reception). As for mild COPD exacerbations, 0 and 84.7% received no and immediate antibiotic prescriptions, respectively, and 4.2% received DAP (directly in all cases).

**Fig. 3:**
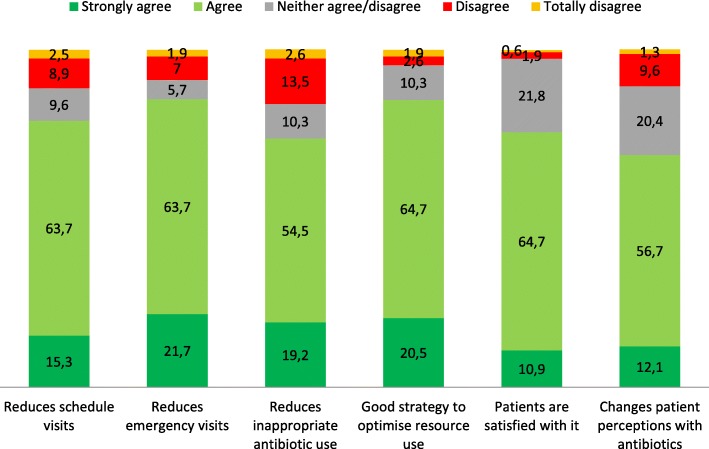
Healthcare professional perceptions of delayed antibiotic prescription

Strongly agree/agree responses from the survey participants (Fig. 3) were as follows: DAP reduces the number of primary-care emergency visits (85.4%; *n* = 134); DAP reduces the number of scheduled visits (79%; *n* = 124); DAP is a good strategy to optimize the use of available resources (85.2%; *n* = 133); DAP reduces inappropriate antibiotic use (73.7%; *n* = 115); patients were satisfied with DAP (75.6%; *n* = 118); and DAP can change patients’ perceptions about the need for antibiotics for certain infections (68.8%; *n* = 108).

Professionals who had already used DAP in routine practice had a consistently more favorable perspective on DAP, as these strongly agreed/agreed more frequently than those who had not used DAP, as follows: DAP reduces the number of primary-care emergency visits (93.6% versus 80%; *p* < 0.001); DAP is a good strategy to optimize the use of available resources (95.1% versus 78.9%; *p* < 0.001); DAP reduces inappropriate antibiotic use (86.9% versus 65.2%; *p* < 0.001)); patients were satisfied with DAP (91.8% versus 65.3%; *p* < 0.001); and DAP can change patients’ perceptions about the need for antibiotics for certain infections (87.1% versus 56.9%; *p* < 0.001). Differences were only non-significant for DAP reducing the number of scheduled visits (80.7% versus 77.9%; *p* = 0.131).

Factors that reflected significantly higher rates of DAP use were as follows: participation versus non-participation in the DAP RCT (74.1% versus 46.2%; *p* < 0.001), having previously used versus not having previously used DAP (86.8% versus 44.2%; *p* < 0.001), and being a physician versus being a nurse (81.8% versus 18.2%; *p* < 0.001). No significant differences in DAP use were observed in relation to the following factors: having rapid diagnostic techniques available; age (mean 46.7 versus 46.4 years; *p* = 0.796); work experience (mean 21.8 versus 21.71 years; *p* = 0.929); and employment in a teaching center versus a non-teaching center (69.2% versus 65%; *p* = 0.561).

## Discussion

### Main findings

Our study shows that an important proportion of primary healthcare professionals make no use of DAP strategies for the treatment of acute uncomplicated RTIs. DAP, when used, was most frequently used for pharyngotonsillitis and least frequently used for otitis and sinusitis. Professionals who became aware of DAP during the RCT started to implement a DAP strategy in their own routine clinical practice.

Most of the respondents considered DAP to reduce the number of primary-care emergency visits (85.4%), the number of scheduled visits (79%), and inappropriate antibiotic use (73.7%), and most also considered that patients were broadly satisfied with DAP (75.6%). Use of DAP was not affected by the fact of having rapid diagnostic techniques available, age, work experience or the fact of being employed in a teaching versus a non-teaching center.

### Our results in the context of previous research

The level of use of DAP as documented in our study (46%) is lower than in northern European countries; a Norwegian study [15], for instance, reported that almost 70% of family physicians considered DAP to be a feasible strategy for treating uncomplicated RTIs. According to that study, sinusitis was the infection for which DAP was most used, contrasting with our study, in which DAP was most frequently used for pharyngotonsillitis.

Although our results show lower use of DAP in Spain than in English-speaking countries, noteworthy is the fact its use led to more positive perceptions of DAP. This finding resonates with results from other countries with a lengthy DAP track record [16]. Note, however, that a qualitative study conducted in the UK showed that DAP was not considered to be a feasible strategy by physicians, as these felt uncomfortable giving patients clinical responsibilities, and only used it for uncertain diagnoses or to avoid conflict with patients [17]. This would indicate that it is important to determine the baseline situation of a country before designing, disseminating, and implementing DAP strategies in routine practice. This was done, for instance, in Australia [18], where, in an effort to combat high antibiotic prescription rates, strategies, including DAP, were designed and implemented to reduce inappropriate antibiotic use.

Spain continues to have a particularly high rate of antibiotic prescription [19]; moreover, the latest update on antibiotic use published by the European Center for Disease Prevention and Control —referring to the period 2010–2014— pointed to an increasing trend in the European Union in general [20]. High antibiotic prescription rates not only represent an economic burden but are also a serious public health problem, since overuse of antibiotics is the main cause of antimicrobial resistance. The latest data for the European Union confirm that a growing number of patients are infected by resistant bacteria [21].

Patients are not generally aware of the serious implications of antimicrobial resistance, nor are they aware that they too can contribute to the solution [22]. Although the association between antibiotic prescription and antimicrobial resistance is well documented, studies show that reduced antibiotic prescription at the primary care level can help reduce antibiotic resistance [23]. Evidence-based strategies are needed to reduce inappropriate antibiotic use in primary care settings, and DAP is one such strategy that has been shown to be highly effective [6, 13]. The absence of information on DAP use in Spain motivated us to conduct this study.

### Limitations and strengths

The main limitation of our study was the low response rate to the survey despite several reminders. Another limitation was that we exclusively surveyed professionals from healthcare centers where the DAP RCT was conducted. Thus, since some non-participants in that RCT may have become aware of DAP through word-of-mouth, our results on DAP use may be overestimated. Nonetheless, this fact merely strengthens our conclusions.

The main strengths of our study are that, as far as we know, this is the first Spanish multicenter survey (23 participating centers) exploring use of DAP among healthcare professionals, and evaluating predictive factors regarding DAP use. Another potential strength is that we also included nurses in the survey; given that they prescribe symptomatic treatment and participate in DAP procedures.

### Implications for practice and research

The current level of use of DAP by healthcare staff in Spain suggests that much needs to be done to make this strategy known among primary care health professionals. Healthcare policymakers should also be made aware of DAP as a potentially effective way to improve decision-making regarding antibiotics and to rationalize their prescription and use in primary care settings. It is also important to foster awareness of DAP as a potential treatment strategy among patients. In order to achieve this we should make known to the GP both the results obtained in other countries, and the excellent results that were obtained in our own country without forgetting beforehand to address the barriers that we could find for Implement the DAP in the usual GP practice as well as the barriers that patients can offer to accept them. Further studies of optimal strategies for implementing DAP in primary care, both in Spain and elsewhere. Thus qualitative research is necessary, which will reveal the barriers that we can find for its implementation by both sides, professionals and patients. As well as it will also provide us with information about the perspectives of the patients and how they receive the DAP and how they use. In this way our group are conducting a qualitative research study in parallel to assess these items, with groups of both professionals and patients.

## Conclusions

Most primary care professionals in Spain still do not use DAP in routine practice. Once professionals become aware of and use DAP, they report that this strategy reduces primary care scheduled and emergency visits and inappropriate antibiotic use, while maintaining patient satisfaction. These findings, combined with positive efficacy and safety results from clinical studies of DAP, highlight the need to actively implement this strategy in primary care.

## Additional files

Additional file 1: Search strategy. Search strategy in MEDLINE (via PubMed) from inception up to March 2012. (PDF 12 kb)

Additional file 2: Delayed antibiotic prescription (DAP) questionnaire. This is a survey aimed at understanding perceptions and attitudes of primary care professionals to antibiotic prescription for uncomplicated infections. (PDF 35 kb)

## Abbreviations

COPD: Chronic obstructive pulmonary disease
DAP: Delayed antibiotic prescription
RCT: Randomized controlled trial
RTI: Respiratory tract infection
