# Supplementary material for: Use of delayed antibiotic prescription in primary care: a cross-sectional study
Source: BMC Fam Pract. 2019 Mar 26;20:45. doi: 10.1186/s12875-019-0934-7 (PMC6434640; doi:10.1186/s12875-019-0934-7)
Supplement: Supplementary file 1 — Search strategy. Search strategy in MEDLINE (via PubMed) from inception up to March 2012. (PDF 12 kb) [file 12875_2019_934_MOESM1_ESM.pdf]

Search strategy in MEDLINE (via PubMed) from inception up to March 2012.

#1 Search delayed prescribing[tiab]	21
#2 Search wait-and-see-prescription*[tiab]	6
#3 Search delayed antibiotic*[tiab]	56
#4 Search delayed antibiotic prescription[tiab]	*4*
#5 Search "deferred prescribing"	*6*
#6 Search "delay of antibiotic prescription*"	*35*
**#7 Search #5 OR #4 OR #3 OR #2 OR #1 OR #6**	**112**
#1 Search "delay of antibiotic prescription*"	35
#2 Search "deferred prescribing"	
#3 Search delayed antibiotic*[tiab]	56
#4 Search wait-and-see-prescription*[tiab]	6
#5 Search delayed prescribing[tiab]	21
#6 Search delayed antibiotic prescription[tiab]	4
#7 Search #6 OR #5 OR #4 OR #3 OR #2 OR #1	112
#8 Search Questionnaire*[tiab]	245943
#9 Search Survey[tiab]	269467
#10 Search #9 OR #8	466887
**#11 Search #7 AND #10**	**8**
