# Supplementary material for: Use of delayed antibiotic prescription in primary care: a cross-sectional study
Source: BMC Fam Pract. 2019 Mar 26;20:45. doi: 10.1186/s12875-019-0934-7 (PMC6434640; doi:10.1186/s12875-019-0934-7)
Supplement: Supplementary file 2 — Delayed antibiotic prescription (DAP) questionnaire. This is a survey aimed at understanding perceptions and attitudes of primary care professionals to antibiotic prescription for uncomplicated infections. (PDF 35 kb) [file 12875_2019_934_MOESM2_ESM.pdf]

Dear colleague

This survey is part of a research project, funded by the Fondo de Investigaciones Sanitarias (FIS; EC08/00095), aimed at knowing perceptions and attitudes of primary care professionals to antibiotic prescription for uncomplicated infections. We would appreciate your collaboration in this study, as it will provide valuable information to all healthcare professionals and the National Health Service in general.

This survey is entirely anonymous and you are free to participate or not. If you choose to respond to the questionnaire, it should take you less than 10 minutes. By completing it you are consenting to the analysis of your results as a whole.

We would like to thank you for your effort and collaboration.

Respondent sociodemographic data

1. Date of birth (DD/MM/YYYY)

   ☐ ☐ / ☐ ☐ / ☐ ☐ ☐ ☐

2. Sex

   ☐ Male

   ☐ Female

3. Healthcare category

   ☐ Physician

   ☐ Medical resident

   ☐ Nurse

4. Year you finished your degree (DD/MM/YYYY)

   ☐ ☐ / ☐ ☐ / ☐ ☐ ☐ ☐

5. Have you completed your PhD thesis?

   ☐ Yes

   ☐ I am currently doing it

   ☐ No

6. Are you currently accredited as a teaching tutor?

   ☐ Yes

   ☐ No

7. In what center are you currently working?

   ☐ Jaume I Care Center, Catalonia

   ☐ Badalona 5 Sant Roc Care Center, Catalonia

   ☐ Badalona 4Gorg Care Center, Catalonia

   ☐ Canet de Mar Care Center, Catalonia

- ☐ Dr. Carles Ribas Care Center, Catalonia
- ☐ Numància Care Center, Catalonia
- ☐ Montnegre Care Center, Catalonia
- ☐ Mataró 6 (Gatassa) Care Center, Catalonia
- ☐ Maria Bernades Care Center, Catalonia
- ☐ Mar Báltico Care Center, Madrid
- ☐ Vicente Muzas Care Center, Madrid
- ☐ Virgen del Cortijo Care Center, Madrid
- ☐ Monovar Care Center, Madrid
- ☐ Ángela Uriarte Care Center, Madrid
- ☐ Mendiguchía Carriche Care Center, Madrid
- ☐ El Soto Care Center, Madrid
- ☐ General Ricardos, Care Center, Madrid
- ☐ Las Américas Care Center, Madrid
- ☐ Irurtzun Care Center, Navarra
- ☐ Huarte Care Center, Navarra
- ☐ Iturrama Care Center, Navarra
- ☐ Alza Care Center, Basque Country
- ☐ Zarautz Care Center, Basque Country

8. Are you currently working in a teaching center?

- ☐ Yes
- ☐ No

9. What point-of-care tests do you currently use at your center?

- ☐ CRP

☐ Rapid antigenic detection tests: Strep-A

☐ Urine dipsticks

☐ I don't know

☐ Other

- Please specify ____

Clinical scenarios

We would like to know which strategy you would consider the most suitable for the following clinical scenarios.

You are in consultation with Claudia, a 23-year old healthy woman complaining of a sore throat, fever (axillary temperature 38.3ºC) that started 24 hours previously, but no cough. The physical examination reveals a hyperemic pharynx without exudate and tender cervical nodes.

10. What strategy would you consider best for this case?

☐ Immediate antibiotic prescription.

☐ Delayed antibiotic prescription, patient-led (the patient receives a prescription but is told to only obtain the antibiotic from their pharmacist and take it if their condition worsens or fails to improve, taking into account the natural history of the infection).

☐ Delayed antibiotic prescription, collected from reception (the patient is told that a prescription can be collected from reception in 48-72 hours if their condition worsens or fails to improve, taking into account the natural history of the infection).

☐ No antibiotic prescription.

☐ Other

- Please specify: ____

You are in consultation with Juan, a 77-year old smoker diagnosed with COPD, with a latest recorded FEV_1 of 45%, presenting with fever (axillary temperature 38.5ºC), cough, and mucopurulent expectoration that started 2 days previously.

11. What strategy would you consider best for this case?

- ☐ Immediate antibiotic prescription.
- ☐ Delayed antibiotic prescription, patient-led (the patient receives a prescription but is told to only obtain the antibiotic from their pharmacist and take it if their condition worsens or fails to improve, taking into account the natural history of the infection).
- ☐ Delayed antibiotic prescription, collected from reception (the patient is told that a prescription can be collected from reception in 48-72 hours if their condition worsens or fails to improve, taking into account the natural history of the infection).
- ☐ No antibiotic prescription.
- ☐ Other
  - o Please specify: ____

Awareness of and participation in the DAP RCT

Your center is participating in a randomized controlled trial (RCT) on DAP for uncomplicated acute respiratory tract infections in adults.

12. Are you aware of the DAP RCT underway in your center?

- ☐ Yes
- ☐ No
- ☐ I don’t know

13. Are you participating in the DAP RCT?

- ☐ Yes
- ☐ No
- ☐ I don’t know

14. Did you know what DAP was before the DAP RCT?

- ☐ Yes
- ☐ No
- ☐ I don’t know

DAP definition

Delayed antibiotic prescription (DAP) is a strategy that consists of issuing an antibiotic prescription to a patient and asking them to only use it if their conditions worsens or fails to improve after a given number of days (depending on the infection). This prescription can be issued at the visit (patient-led) or can be collected, if needed, from the center reception.

Use of DAP

15. As a physician or nurse, did you use or recommend DAP in routine practice before the RCT?

☐ Yes

☐ No

☐ I don't know

16. As a physician or nurse, are you currently using DAP in routine practice?

☐ Yes

☐ No

☐ I don't know

17. If, as a physician or nurse, you have used or recommended DAP in routine practice, for what type of infections have you done so? (you may choose more than one option)

Conditions	Yes	No
**Respiratory**	☐	☐
**Urinary**	☐	☐
**Digestive**	☐	☐
**Skin**	☐	☐
**Eye**	☐	☐
**Dental**	☐	☐
**Other**	☐	☐

o If other, please specify ____

18. If, as a physician or nurse, you have used or recommended DAP in routine practice for respiratory conditions, for what specific conditions have you done so? (you may choose more than one option)

Condition	Yes	No
**Pharyngotonsillitis**	☐	☐
**Acute sinusitis**	☐	☐
**Acute otitis media**	☐	☐
**Acute bronchitis**	☐	☐
**COPD exacerbation**	☐	☐
**Acute cystitis**	☐	☐
**Acute conjunctivitis**	☐	☐
**Dental infection**	☐	☐
**Other**	☐	☐

- If other, please specify ____

20. If, as a physician or nurse, you are currently using DAP in routine practice, what DAP strategy do you use?

☐ I refer the patient to their physician if the condition worsens or fails to improve.

☐ I issue a prescription to the patient to be used in the event that their condition worsens or fails to improve, taking into account the natural history of the infection.

☐ I issue a prescription and leave it at reception for the patient to collect if, in 24-48 hours, their condition worsens or fails to improve, taking into account the natural history of the infection.

☐ Other

- Please specify ____

Perceptions of DAP

21. Do you think that using DAP …

	Totally disagree	Disagree	Neither agree/disagree	Agree	Totally agree
**Can reduce subsequent scheduled visits?**	☐	☐	☐	☐	☐
**Can reduce subsequent emergency visits to your health center?**	☐	☐	☐	☐	☐
**Can reduce inappropriate antibiotic use for some infections?**	☐	☐	☐	☐	☐
**Could be a good strategy for optimizing available resource use?**	☐	☐	☐	☐	☐

22. Do you think that patients …

	Totally disagree	Disagree	Neither agree/disagree	Agree	Totally agree
**Can influence their physicians/nurses in using/recommending DAP?**	☐	☐	☐	☐	☐
**Are satisfied if they have used DAP?**	☐	☐	☐	☐	☐
**Change their perceptions of the need for antibiotics to treat certain infections if they have used DAP?**	☐	☐	☐	☐	☐
